# Intra‐Operative Onychoscopy of Subungual Epidermoid Inclusions

**DOI:** 10.1111/1346-8138.17825

**Published:** 2025-06-13

**Authors:** Grigorios Theodosiou, Peter C. Schalock, Åke Svensson

**Affiliations:** ^1^ Department of Dermatology Skåne University Hospital Malmö Sweden; ^2^ Department of Clinical Sciences Lund University Lund Sweden

**Keywords:** dermoscopy, intraoperative onychoscopy, nail disorders, nail surgery, onychoscopy, subungual onycholemmal cysts

## Abstract

Subungual epidermoid inclusions (SEI) are small epithelial inclusions, sometimes solid but mostly with central keratinization of the onycholemmal type without a granular layer. Exceptionally, they become large enough to produce symptoms such as pain or swelling of the nail bed, resulting in subungual keratosis, onycholysis, or a dystrophic nail plate. Herein, we report a further case of SEI in a female patient with onychodystrophy of the great toe nail and highlight the onychoscopic features of SEI. Intra‐operative onychoscopy facilitates the detection of SEI during a nail bed biopsy and reduces the risk of obtaining inadequate specimens for histopathologic examination.

## 1 Introduction

Subungual epidermoid inclusions (SEI), also known as subungual onycholemmal cysts, represent a distinctive nail disorder arising from the nail bed. They are small epithelial inclusions, sometimes solid but mostly with central keratinization of the onycholemmal type without a granular layer. SEI usually remain microscopic and asymptomatic. Exceptionally, they become large enough to produce symptoms such as swelling of the nail bed resulting in subungual keratosis, onycholysis, or a dystrophic nail plate. Initially, this is painless but later mild pain may appear due to compression between nail and bone. The nail plate may be normal or dystrophic; thickened, clubbed or ridged.

## 2 Case Report

A 50‐year‐old Caucasian woman was referred with onychodystrophy of the right great toe nail. The patient had lifelong atopic dermatitis and was otherwise healthy. She observed nail pigmentation and progressive thickening of her left great toe nail 2 years before the referral. She could not recall any trauma and had no local symptoms. There was no family history of melanoma or other skin malignancies. Fungal studies were previously negative.

**FIGURE 1:**
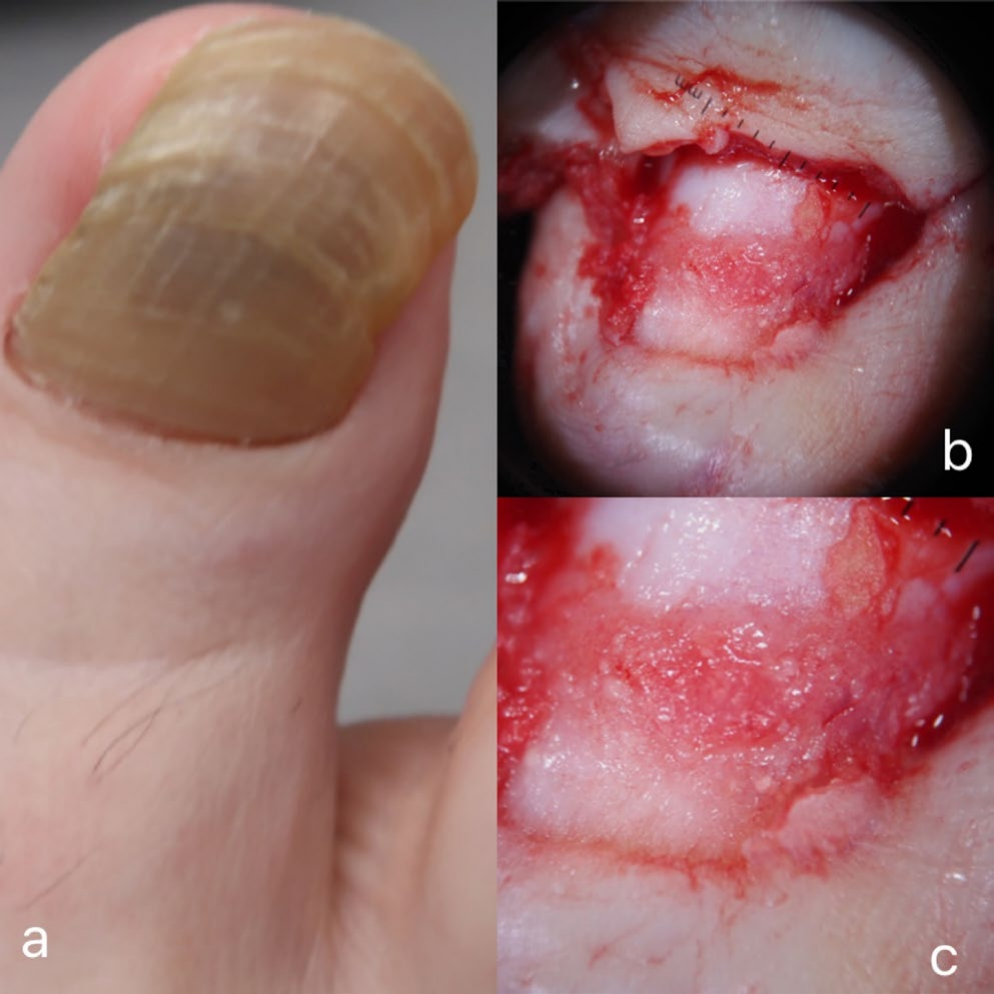
(a) Diffuse nail plate thickening, brownish discoloration, and marked lateral malalignment of the right great toenail. (b) Intraoperative polarized contact onychoscopy (magnification ×10). (c) Whitish dome‐shaped projections on the nail bed (magnification ×20).

Clinical examination of the right great toenail revealed diffuse nail plate thickening, brownish discoloration and marked lateral malalignment (Figure 1a) The remaining toenails and fingernails were unaffected. A complete nail avulsion was performed for exploration and nail bed biopsy. Grossly, the nail bed appeared slightly edematous with multiple scattered 1–2 mm skin‐colored to whitish dome‐shaped projections, a finding more obvious with intraoperative polarized contact onychoscopy performed through a dermatoscope DermLite Foto X (3Gen, San Juan Capistrano, CA, USA) with the aid of ultrasound gel (Figure 1b,c). No pigmentation of the nail matrix or nail bed was observed. Two 3‐mm punch biopsies were obtained from the nail‐bed.

**FIGURE 2:**
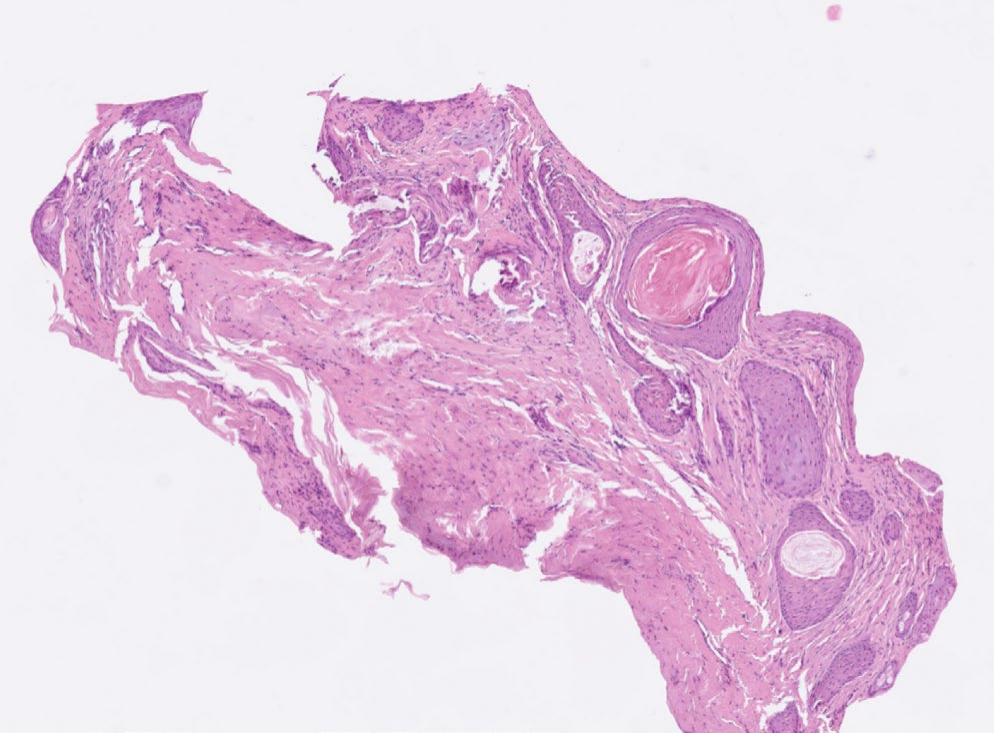
Multiple epidermoid cysts within the dermis of the nail bed, in close proximity to the epithelium (H&E ×10).

Histopathological examination revealed multiple cysts within the dermis of the nail bed, in close proximity to the epithelium. The cysts were lined by stratified squamous epithelium without any cellular atypia. (Figure 2) Immunohistochemical staining with SOX was negative. Postoperatively, the defect healed by secondary intention without any complication.

## 3 Discussion

SEI were first described in 1959 in toe nails by Samman [1]. Later, Lewin reported several examples of SEI in surgical and postmortem specimens of normally appearing or clubbed nails [2]. Apart from their possible relation to trauma and a reported association with finger clubbing, little is known about their etiology. In Lewin's review of 90 consecutive autopsies, epidermoid inclusions were found in all eight cases of nail clubbing and also in many normal nails (the exact number was not reported) [3].

Another case series of eight cases reported the subungual hyperkeratosis with shortened and dystrophic nail plate as the most striking clinical feature. A history of trauma was reported by 4 patients. The thumb and the great toe were most commonly involved (7 out of 8 patients). Onycholysis was observed in one case. After a 2‐year follow‐up most of the lesions remained unchanged [4].

No pathognomonic clinical alternations exist; the nail may look normal or be thickened, dystrophic, ridged or yellow and the nail bed may appear hyperkeratotic. The differential diagnosis includes onychomycosis, squamous cell carcinoma, glomus tumor, subungual melanoma, and other tumors of the nail matrix [5].

A full‐thickness nail‐bed biopsy is required to make the diagnosis because SEI are microscopic rather than macroscopic. Histopathology shows hyperplasia of the nail bed and small epithelial inclusions, sometimes solid but mostly with central keratinization of the onycholemmal type without a granular layer. Secondary calcification is common [6]. The relationship of calcified onycholemmal cysts to the subungual calcification in elderly persons is not yet clear. Sometimes, the onycholemmal cysts are seen to derive from the elongated rete ridges of the nail bed epithelium. Once the diagnosis has been made, no further treatment is needed. No treatment is curative and simply making the diagnosis prevents inappropriate treatments and relieves anxiety over possible malignancies [7].

Onychoscopy of the nail unit is a non‐invasive method that allows observation of the nail structures, increasing the accuracy of clinical diagnosis. Intra‐operative onychoscopy is a variant of this technique which enables direct visualization of the nail unit structures after avulsion of the nail plate [8]. Although intra‐operative onychoscopy does not replace histopathologic examination, it may be useful for intra‐operative evaluation of suspect lesions that require pathologic examination in order to select the most appropriate biopsy site or to determine what type of biopsy should be performed. Improperly planned or blind nail bed biopsies carry the risk of obtaining an inadequate specimen and may miss the lesion of concern. Application of intra‐operative onychoscopy in our case facilitated the identification of the SEIs and the selection of the biopsy site.

## 4 Conclusion

Herein, we report a further case of SEI in a female patient with onychodystrophy of the great toe nail and highlight the onychoscopic features of SEI. Intra‐operative onychoscopy facilitates the detection of SEI during a nail bed biopsy and reduces the risk of obtaining inadequate specimens for histopathologic examination.

## Conflicts of Interest

The authors declare no conflicts of interest.

## Data Availability

The data that support the findings of this study are available from the corresponding author upon reasonable request.
